# Cardia function-preserving surgery and anti-reflux anastomotic method after proximal gastrectomy for gastric cancer: Current status and future perspectives

**DOI:** 10.3389/fonc.2022.1000719

**Published:** 2022-12-15

**Authors:** Li Li, Zheng-hui Liu, Xu-fan Cai, Qi-tao Jiang, Yi-ping Mou, Yuan-Yu Wang

**Affiliations:** ^1^ Department of General Surgery, Cancer Center, Division of Gastrointestinal and Pancreatic Surgery, Zhejiang Provincial People’s Hospital, Affiliated People’s Hospital, Hangzhou Medical College, Hangzhou, Zhejiang, China; ^2^ Key Laboratory of Gastroenterology of Zhejiang Province, Hangzhou, Zhejiang, China; ^3^ Bengbu Medical College, Bengbu, Anhui, China; ^4^ Zhejiang Chinese Medical University, Hangzhou, Zhejiang, China

**Keywords:** cardia function-preserving, surgery, anti-reflux anastomotic, gastric cancer, proximal gastrectomy

## Abstract

The incidence and mortality of gastric cancer ranked 5th and 3rd worldwide, respectively, in 2018, and the incidence of gastroesophageal junction adenocarcinoma increased over the past 40 years. Radical resection and lymph node dissection is the preferred treatment for gastric cancer. Proximal gastrectomy or total gastrectomy is usually performed for gastroesophageal junction adenocarcinoma and upper gastric cancer. Owing to the resection of the cardia structures, the incidence of reflux esophagitis increases significantly after proximal gastrectomy and total gastrectomy, resulting in poor postoperative quality of life. To reduce the incidence of reflux esophagitis and improve patients’ postoperative quality of life, various methods to preserve the function of the cardia or to perform anti-reflux reconstruction have emerged. In this manuscript, we systematically introduced the advantages and problems of various anti-reflux anastomotic method after proximal gastrectomy, and cardia-preserving gastrectomy including endoscopic resection (ER), local gastrectomy by gastroscopy combined with laparoscopy, segmental gastrectomy, subtotal gastrectomy, and cardia-preserving radical gastrectomy. Cardia-preserving radical gastrectomy has the advantage of more thorough lymph node dissection and wider indications than those for subtotal gastrectomy. However, the clinical efficacy of cardia-preserving radical gastrectomy requires verification in prospective and controlled clinical trials. Cardia-preserving radical gastrectomy is a promising approach as one of the more reasonable anti-reflux surgeries.

## 1 Introduction

The incidence and mortality of gastric cancer ranked 5th and 3rd worldwide, respectively, in 2018, and the respective rates in China were 44.1% and 49.9% (1). In 2015, data from the National Cancer Center of China showed that the incidence of gastric cancer in men and women ranked 2nd and 3rd, respectively, and mortality ranked 3rd (2). Data from the Surveillance, Epidemiology, and End Results (SEER) database of the National Cancer Institute indicated that the incidence of gastroesophageal junction adenocarcinoma increased 2.5-fold over the past 35 years (3). Data from the National Cancer Center Japan showed that the incidence of gastroesophageal junction adenocarcinoma increased 7.3% from the 1960s to the early 2000s (4). In China, the incidence increased from 22.3% in 1988 to 35.7% in 2012 (5).

Radical resection and lymph node dissection is the preferred treatment for gastric cancer. Proximal gastrectomy or total gastrectomy is usually performed for gastroesophageal junction adenocarcinoma and upper gastric cancer. Anatomically, the cardia is the opening between the esophagus and the stomach, with the junction of the stomach and the esophagus as an initial segment, and the cardia is connected to the lower segment of the esophagus. There is a 2–3 cm long thickened and hypertrophic annular muscle layer in the lower esophagus containing the distributions of the spinal nerves and the vagus nerve that constitutes the lower esophageal sphincter. The sphincter mainly maintains the lower intraesophageal pressure at rest (15–30 mmHg higher than the intragastric pressure), and the sphincter can generate a pressure of approximately 100 mmHg during persistent contraction (6, 7). Owing to the resection of the cardia structures, the incidence of reflux esophagitis increases significantly after proximal gastrectomy and total gastrectomy (14.5% and 5.4%, respectively), resulting in poor postoperative quality of life (8). To reduce the incidence of reflux esophagitis and improve patients’ postoperative quality of life, various methods to preserve the function of the cardia or to perform anti-reflux reconstruction have emerged. This manuscript mainly discusses the importance of preserving cardiac function, including two parts: reservation of cardia and resection of cardia.

## 2 Anti-reflux anastomotic method after proximal gastrectomy for gastric cancer

In first part of resection of cardia, proximal gastrectomy preserves partial stomach function but results in the loss of the anti-reflux function of the cardia, and the preserved pylorus delays gastric emptying to some extent (**Table 1**) (14). Thus, severe reflux esophagitis occurs easily after proximal gastrectomy (8). Recently, various methods of anti-reflux digestive tract reconstruction after proximal gastrectomy have emerged, which not only preserve partial gastric function, but also avoid severe reflux esophagitis.

**Table 1:** Anti-reflux anastomotic method after proximal gastrectomy for gastric cancer.

Number	Anastomotic method	Time of first report	Disadvantage
1	Gastroesophagostomy		
1.1	Tubular gastroesophagostomy	1998, Shiraishi (9)	the incidence of reflux symptoms and anastomotic stenosis was higher
1.2	Side overlap anastomosis	2016, Yamashita (10)	retention of a long abdominal esophagus and a large remnant stomach (more than 2/3)
1.3	Double⁃flap anastomosis (Kamikawa anastomosis)	1998, Kamikawa (11)	double-flap technique is complicated and requires advanced suturing skills and a long operative time
2	Jejunal interposition	1993, Kameyama (12)	the operation is complicated, with a long operative time and relatively high cost, and there is the possibility of obstructed remnant stomach emptying
3	Double-tract reconstruction (DTR)	1988, Aikou (13)	the surgical procedure is relatively complicated, with many anastomotic stomas, possibly increasing the risk of stomal leakage and increased costs

A type of proximal gastrectomy and piggyback jejunal interposition has been reported to block the jejunum at the distal end of the gastrojejunal anastomosis. This approach is based on double-tract anastomosis, which is a continuous interjejunal anastomosis. This procedure preserves the continuity of the interpositioned jejunal segment, reduces the possibility of obstructed food emptying, and improves the patient’s nutritional status (10, 30).

### 2.1 Gastroesophagostomy

#### 2.1.1 Tubular gastroesophagostomy

**Figure 1:**
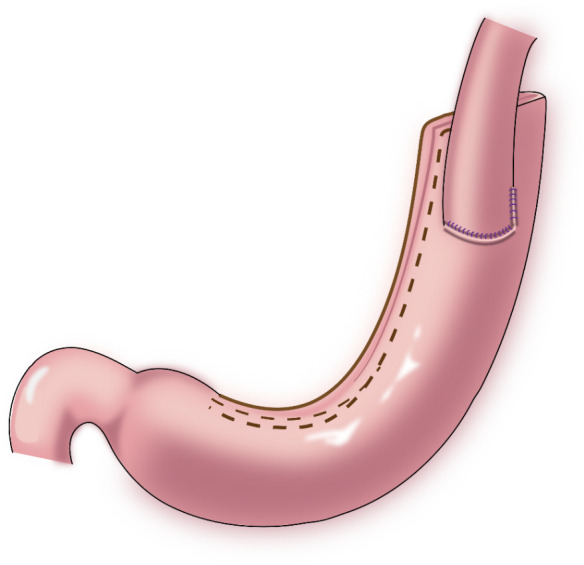
Tubular gastroesophagostomy: the esophagus was anastomosed with the anterior wall of the remnant stomach by which a fundus-like structure is created at the top of the remnant stomach.

**Figure 2:**
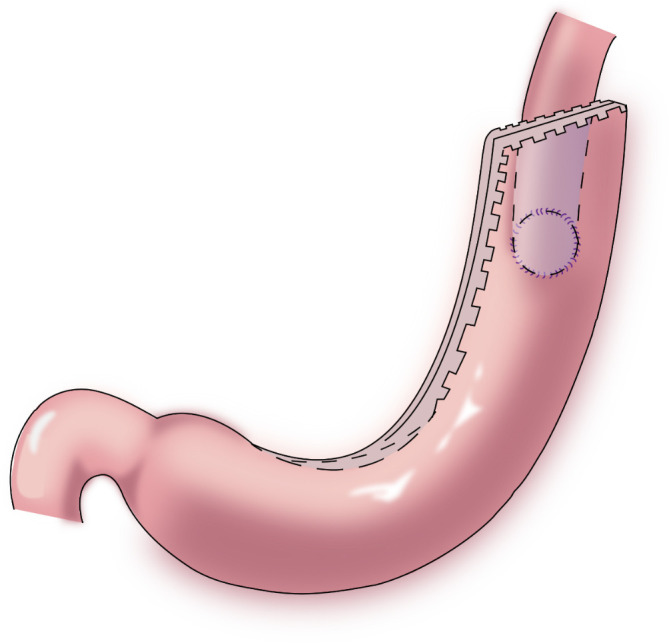
Tubular gastroesophagostomy: the esophagus was anastomosed with the posterior wall of the residual stomach using a tubular stapler.

In 1998, Shiraishi et al. first reported tubular gastroesophagostomy, by which a fundus-like structure is created at the top of the remnant stomach (**Figure 1**) (15). The regurgitated gastric juice is temporarily stored in the “fundus” when patients are in a supine position, avoiding direct reflux to the lower end of the esophagus to some extent. Part of the gastric antrum is resected from the tubular stomach in this procedure, which reduces the secretion of gastrin and gastric acid. The tubular stomach maintains the anatomical structure of the stomach, leading to a higher quality of life than that of patients undergoing traditional anastomosis of the gastric remnant to the esophagus (16). Chen et al. found that only 14.3% of the patients undergoing tubular gastroesophagostomy presented with reflux symptoms postoperatively, and 5.7% of these patients were diagnosed with reflux esophagitis (17). Additionally, the degree of reflux esophagitis after tubular gastroesophagostomy was lower than that with traditional anastomosis of the gastric remnant to the esophagus (17). Ronellenfitsch et al. reported that 30% of patients experienced reflux symptoms after tubular gastroesophagostomy, but that symptoms were mild in all patients (18). Kukar et al. reported that 6 case of tubular gastroesophagostomy, the esophagus was anastomosed with the posterior wall of the residual stomach using a tubular stapler. All patients had negative final margins and an adequate lymph node dissection (median number of nodes examined was 15, range 12-22). The median postoperative length of stay was 7 days (range 4-7). Two patients developed anastomotic strictures requiring intervention, and 1 patient experienced significant reflux. At a median follow-up of 11 months, there was 1 recurrence. Three patients were alive without evidence of disease, and 2 patients died from other causes (**Figure 2**) (19). Aihara et al. reported that the incidence of reflux symptoms after tubular gastroesophagostomy was 16.7%; however, anastomotic stenosis occurred in 35% of the patients (20). Clipping of the tubular stomach is usually performed with a linear cutting stapler, which has a relatively high cost. However, the length of the tubular stomach is longer, and the method is especially suitable for patients with a higher esophageal margin.

#### 2.1.2 Side overlap anastomosis

**Figure 3:**
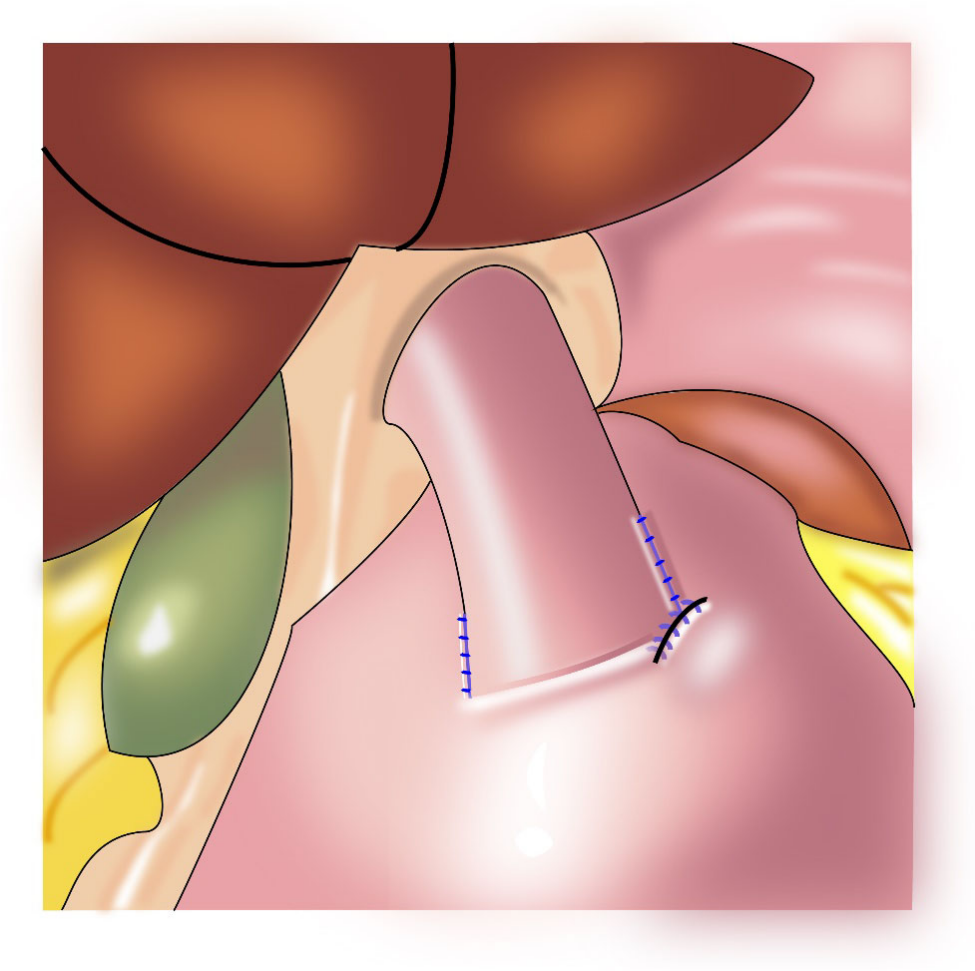
Esophagogastric side-to-side anastomosis: the opposite wall of the esophagus is fixed to the stomach to bring the esophagus close to the stomach wall.

Yamashita et al. first reported side overlap anastomosis in 2016, which generally requires retaining the abdominal esophagus and 2/3 of the remnant stomach. The remnant stomach is fixed at the base of the left and right diaphragm to construct an artificial stomach fundus. Then, esophagogastric side-to-side anastomosis (**Figure 3**) is performed, and the opposite wall of the esophagus is fixed to the stomach to bring the esophagus close to the stomach wall. When the pressure in the artificial fundus increases, the anastomotic stoma closes, which provides an anti-reflux effect (21). The incidence of reflux esophagitis after side overlap anastomosis is 10%, with a wide anastomotic stoma leading to a reduced incidence of anastomotic stenosis (21, 22). The advantages of this procedure are that it is a relatively simple operation, and it is associated with a short anastomosis time and low cost. The disadvantage is the need for retention of a long abdominal esophagus and a large remnant stomach (more than 2/3); therefore, application of this procedure is limited.

#### 2.1.3 Double⁃flap anastomosis (Kamikawa anastomosis)

**Figure 4:**
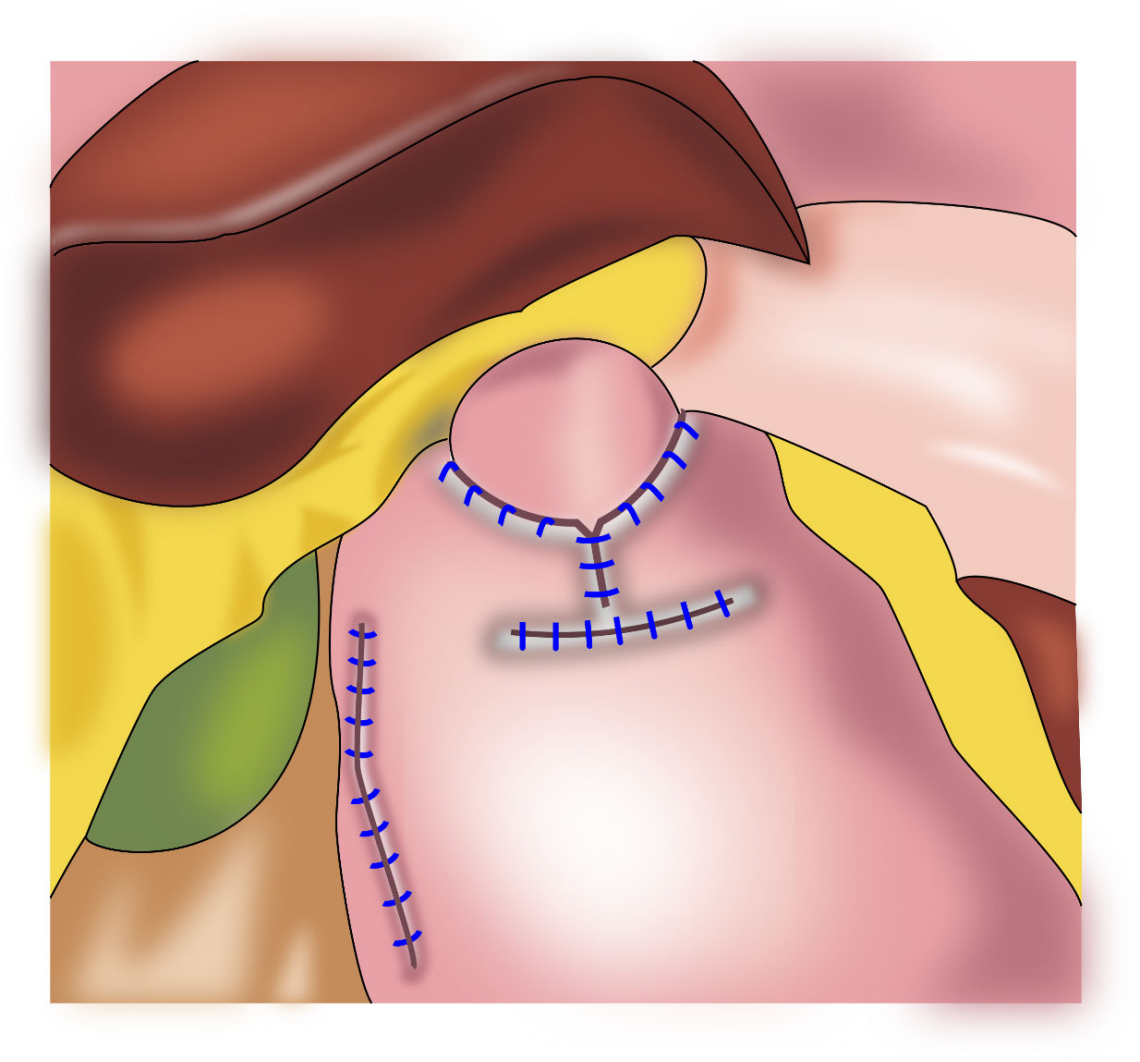
Double⁃flap anastomosis (Kamikawa anastomosis): This procedure increases the pressure in the lower esophagus and is beneficial to reduce the occurrence of reflux esophagitis.

In 1998, Kamikawa reported double-flap esophagogastrostomy (**Figure 4**) to prevent reflux (23), during which a “Gong” (a Chinese character)-shaped seromuscular flap is made below the resection margin of the remnant stomach. At the lower margin of this “window,” the mucosa and submucosa are cut and anastomosed to the esophageal cut margin. Finally, the two seromuscular flaps cover the lower segment of the esophagus and the upper part of the anastomotic stoma. This procedure increases the pressure in the lower esophagus and is beneficial to reduce the occurrence of reflux esophagitis. A multi-center retrospective study from Japan evaluating the efficacy and safety of the double-flap technique included 546 patients from 18 centers, of whom 464 patients underwent endoscopic evaluation of reflux esophagitis 1 year postoperatively. Grade B or higher reflux esophagitis was found in 6% of the patients under endoscopy, and the incidence of anastomotic stenosis was 5.5% (24). This surgical procedure may increase the occurrence of anastomotic stenosis; however, if the width of the seromuscular flaps is appropriately extended, the incidence of anastomotic stenosis may decrease (25). Kuroda et al. believed that the double-flap technique is promising as one of the preferred techniques for digestive tract reconstruction after proximal gastrectomy (24, 26). The double⁃flap technique is suitable for patients with early gastric cancer in the upper third of the stomach with a predicted residual gastric capacity of > 50%. However, the operative procedure for the double-flap technique is complicated and requires advanced suturing skills and a long operative time.

### 2.2 Jejunal interposition

**Figure 5:**
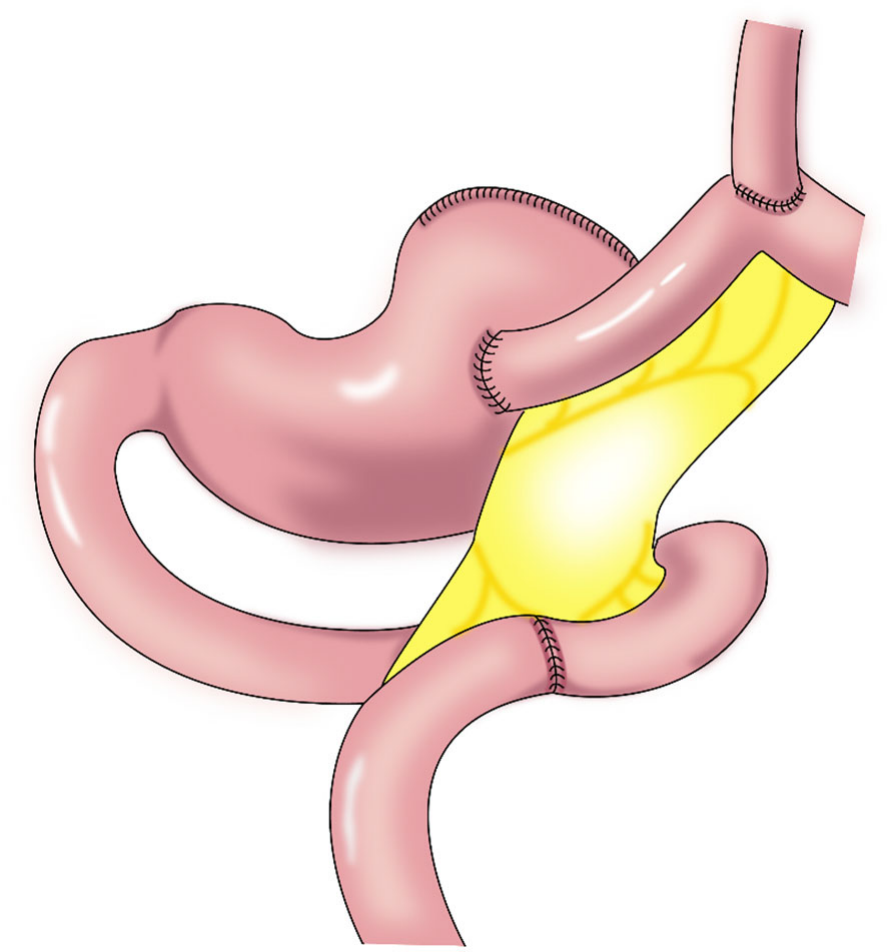
Jejunal interposition: a segment of jejunum is inserted between the esophagus and the remnant stomach to construct an anti-reflux barrier. This procedure takes advantage of intestinal peristalsis and the tolerance of the jejunum to acidic gastric juice and alkaline digestive juice.

In jejunal interposition (**Figure 5**), a segment of jejunum is inserted between the esophagus and the remnant stomach to construct an anti-reflux barrier. This procedures takes advantage of intestinal peristalsis and the tolerance of the jejunum to acidic gastric juice and alkaline digestive juice. Kameyama et al. first reported that the interposition of a jejunal pouch could preserve storage capacity in the remnant stomach (9). Katai et al. reported that the incidence of reflux symptoms after jejunal interposition was 5.6%, and that of reflux esophagitis on endoscopy was 1.7%, which significantly improved the patients’ postoperative quality of life (27). As a disadvantage, food residues are easily retained in the jejunal pouch and remnant stomach (28). To improve this situation and minimize the incidence of reflux esophagitis, the length of the jejunal pouch has been gradually shortened to the current length of approximately 10 cm (29). The small intestine replaces the upper part of the stomach. However, compared with the stomach, the jejunal pouch has thinner fascia and limited storage capacity, which is attributed to histological differences.

The jejunal interposition has a low requirement regarding the remnant stomach size and is suitable for most reconstructions after proximal gastrectomy. However, the operation is complicated, with a long operative time and relatively high cost, and there is the possibility of obstructed remnant stomach emptying.

### 2.3 Double-tract reconstruction (DTR)

**Figure 6:**
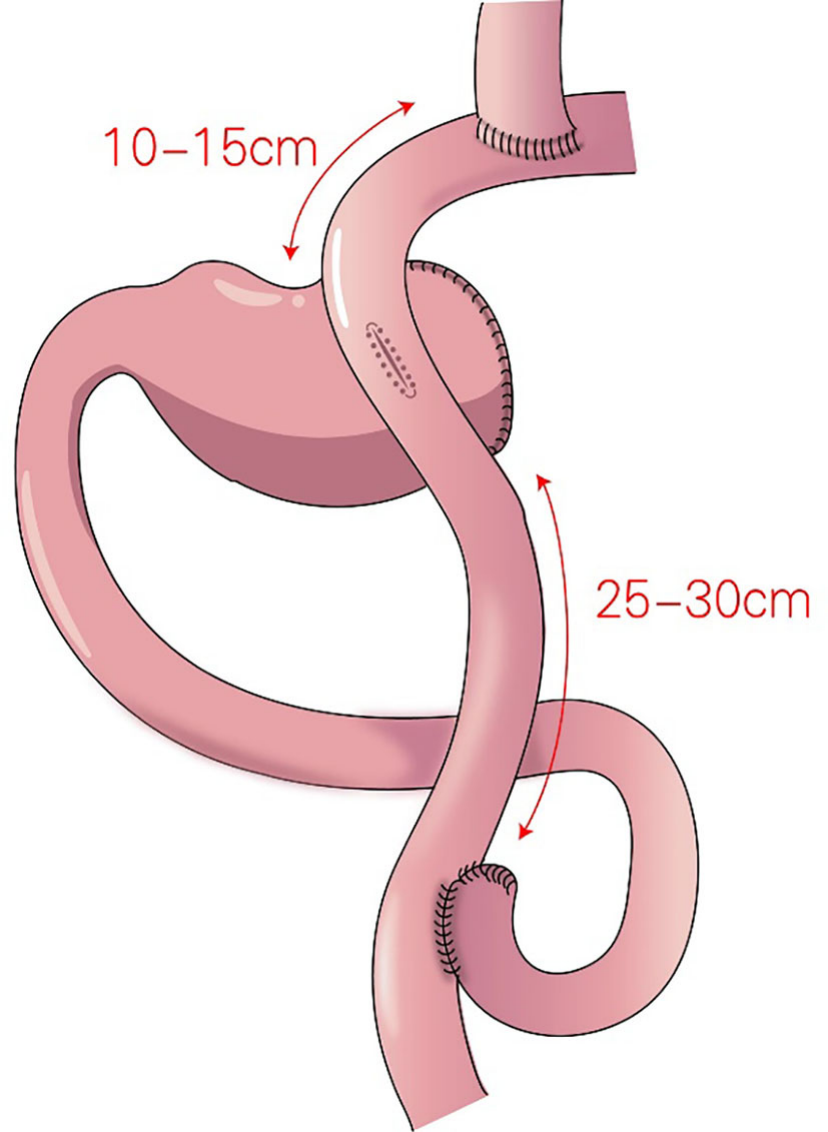
Double-tract reconstruction (DTR): Roux-en-Y anastomosis of the esophagus and jejunum is performed first, the jejunum 10–15 cm from the anastomotic stoma of the remnant stomach, and the esophagus-jejunum are anastomosed side-to-side.

In 1988, Aikou et al. first reported DTR (**Figure 6**) as a type of proximal gastrectomy for digestive tract reconstruction (31). In this method, Roux-en-Y anastomosis of the esophagus and jejunum is performed first, after the proximal stomach is dissociated. Then, the jejunum 10–15 cm from the anastomotic stoma of the remnant stomach, and the esophagus-jejunum are anastomosed side-to-side. After esophagojejunal anastomosis, food can enter the distal jejunum through the remnant stomach and jejunum (31). Nakajima et al. found that DTR with a larger remnant stomach provided better transport and mixing of bile and food, and partial food directly entering the jejunum alleviated slow emptying or food stagnation in the remnant stomach induced by vagotomy (32). Ahn et al. showed that the incidence of reflux esophagitis in the DTR group was 4.6%, indicating a good preventive effect of DTR on reflux symptoms (11). Tomoki et al. reported that the incidences of reflux symptoms (10.5% vs. 54.5%) and anastomotic stenosis (0 vs. 27%) in the DTR group were significantly lower than those in the esophagogastric anastomosis group, respectively, 1 year postoperatively (33). Reo et al. found no difference in early complication rates between the laparoscopic proximal gastrectomy + DTR group and the total gastrectomy group (34). The incidence of reflux esophagitis was significantly higher in the proximal gastrectomy + DTR group than that in the total gastrectomy (Roux-en-Y reconstruction) group (8.0% and 0%, respectively), and the amount of weight loss and the decrease in hemoglobin concentration were significantly lower in the DTR group than in the total gastrectomy group (34).

DTR is appropriate for most reconstructions of the digestive tract after proximal gastrectomy, with low requirements regarding the remnant stomach, and DTR is especially appropriate for patients who require excessive stomach resection and who are not eligible for esophagogastrostomy. However, the surgical procedure is relatively complicated, with many anastomotic stomas, possibly increasing the risk of stomal leakage and increased costs.

## 3 Cardia function-preserving gastrectomy for gastric cancer

In second part of reservation of cardia, we reviewed various methods to preserve the function of the cardia including endoscopic resection (ER), local gastrectomy by gastroscopy combined with laparoscopy, segmental gastrectomy, subtotal gastrectomy, cardia-preserving radical gastrectomy (**Table 2**).

**Table 2:** Function-preserving gastrectomy.

Method of operation	Indications	Disadvantage
Endoscopic resection (ER)	EMR for differentiated-type adenocarcinoma, stage T1a, no ulcerative findings, and tumor diameter ≤ 2 cm. ESD for differentiated-type adenocarcinoma, stage T1a, no ulcerative findings, and no clearly-defined tumor size. ESD for differentiated-type adenocarcinoma, stage T1a combined with an ulcer, with a tumor diameter of ≤ 3 cm.	For patients with postoperative positive resection margins or for those who underwent non-radical resection (such as vascular infiltration) need radical remedial surgery
Local gastrectomy by gastroscopy combined with laparoscopy	Suitable only for early gastric cancer	The indications for surgery are very limited
Segmental gastrectomy	Suitable only for early gastric cancer in the middle third of the stomach, preferably with the cancer located in the greater curvature of the stomach	The indications for surgery are very limited
Subtotal gastrectomy	Suitable only for tumor in the upper stomach or invading the upper stomach, preoperative stage cT1N, tumor located <5cm from the gastroesophageal junction or < 3 cm from the cut end of the remnant stomach, and negative incision margin	Lack of blood supply to the remnant stomach, worsened motility disorders in the remnant stomach, and poor anastomosis healing
Cardia-preserving radical gastrectomy	(1) early gastric cancer, the distance from the upper margin of the lesion to the cardia is ≥4 cm, 2-4 cm can be used as the relative indication. (2) In advanced middle gastric cancer, the incision margin was at least 2.0-3.0 cm distance from the cardia to ensure the anastomotic distance	The number of cases carried out is relatively small, but its clinical efficacy requires further verification in prospective and controlled clinical studies

### 3.1 Endoscopic resection (ER)

Early gastric cancer means that cancer invaded into the mucosa or submucosa, regardless of lymph node metastasis (35). Detection rates of early gastric cancer in Japan and Korea are 70% and 50%, respectively, compared with approximately 20% in China (36).

Endoscopic submucosal dissection (ESD) has been gradually applied to treat early gastric cancer. Isomoto et al. found that the en bloc resection rate in patients with early gastric cancer undergoing ESD was 94.9% (559/589), and the radical resection rate was 94.7% (550/581), The overall 5-year survival rate and disease-specific survival rate were 97.1% and 100%, respectively (12). Thus, ESD achieves a considerable therapeutic effect comparable to that obtained with surgery.

The 5th edition of the Japanese Gastric Cancer Treatment Guidelines recommend endoscopic mucosal resection (EMR) for differentiated-type adenocarcinoma, stage T1a, no ulcerative findings, and tumor diameter ≤ 2 cm. ESD is recommended for differentiated-type adenocarcinoma, stage T1a, no ulcerative findings, and no clearly-defined tumor size. ESD is also recommended for differentiated-type adenocarcinoma, stage T1a combined with an ulcer, with a tumor diameter of ≤ 3 cm. For patients with postoperative positive resection margins or for those who underwent non-radical resection (such as vascular infiltration), radical remedial surgery is recommended (37). The 2021 National Comprehensive Cancer Network guidelines recommend ESD for differentiated-type adenocarcinoma, tumor diameter ≤2 cm, stage T1a, and no lymphatic vascular invasion (38). A study from the UK found that the long-term prognosis of stage T1aN0 and T1bN0 gastric cancer patients undergoing ER was inferior than that of the gastrectomy group (39). However, some studies have found that the survival rate of early gastric cancer patients with lymph node metastasis is significantly lower than that of those without metastasis (40), and the recurrence risk with ESD is higher with lymph node metastasis than without (13, 41, 42).

### 3.2 Local gastrectomy by gastroscopy combined with laparoscopy

Abe et al. first reported endoscopic full⁃thickness resection in the treatment of early gastric cancer in 2008 (43). Full-thickness resection of the gastric wall can achieve vertical and horizontal tumor resection margins that meet the requirements of radical tumor treatment (43). In 2012, using laparoscopy combined with endoscopy, Nunobe et al. performed laparoscopy-assisted full-thickness ER for early gastric cancer with a wide range of lesions, and achieved good effects (44). Hur et al. found that such full-thickness resection by laparoscopy and endoscopy ensured the reliability of the tumor vertical resection margins and that laparoscopy played an important role in lymph node dissection (45). The short-term results of the sentinel node navigation oriented tailored approach from South Korea confirmed that local resection with sentinel lymph node dissection was not inferior to traditional laparoscopic gastrectomy in the treatment of early gastric cancer (46). Local resection is beneficial for preserving gastric function and for achieving better nutritional status and quality of life, but only for early gastric cancer.

### 3.3 Segmental gastrectomy

**Figure 7:**
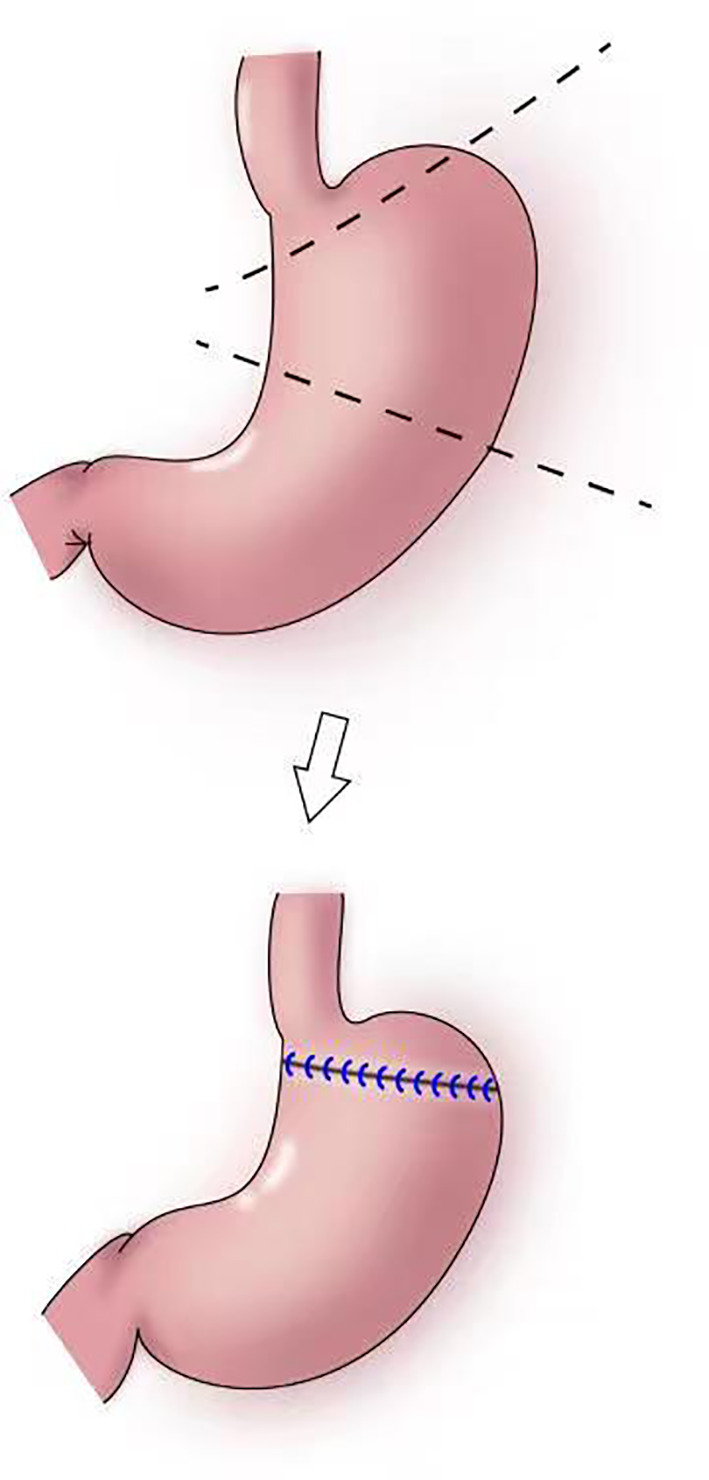
Segmental gastrectomy: The shaded area of the stomach is excised in upper figure. Anastomosis is performed between the distal remnant of the stomach and a fundic pouch in lower figure.

In 1999, Ohwada et al. first used segmental gastrectomy (**Figure 7**) to treat early gastric cancer located in the middle third of the stomach (47). In 2006, Shinohara et al. reported segmental gastrectomy for early gastric cancer in the upper third of the stomach, and found that reflux symptoms and reflux esophagitis were significantly less frequent after segmental gastrectomy compared with those after proximal gastrectomy (48). In 2007, Koichi et al. reported that the incidences of early dumping syndrome and reflux gastritis were significantly lower after segmental gastrectomy compared with those after distal gastrectomy. All patients remained alive without recurrence during a mean follow-up period of 54.7 months in the segmental gastrectomy group (49). In 2010, Takeru et al. reported significantly less reflux esophagitis and reflux gastritis in the segmental gastrectomy group compared with that in the distal gastrectomy group, no recurrence or death was observed in two group following up median of 32.8 months (50). In 2012, Kim et al. proposed cardia-preserving proximal gastrectomy (51), which is a form of segmental gastrectomy. In 2017, Xiao analyzed the efficacy of laparoscopic segmental resection for early gastric cancer, and found no postoperative anastomotic fistulas, gastroparesis, or reflux (52). The number of lymph nodes obtained was 18.3 ± 7.5, and no severe gastroparesis occurred.

The indications for segmental gastrectomy are very limited, and the procedure is suitable only for early gastric cancer in the middle third of the stomach, preferably with the cancer located in the greater curvature of the stomach. Intraoperatively, lymph nodes on the lesser curvature should be resected, and hepatic and abdominal branches of the vagus nerve should be preserved. The surgical skill are also difficulties (52).

### 3.4 Subtotal gastrectomy

**Figure 8:**
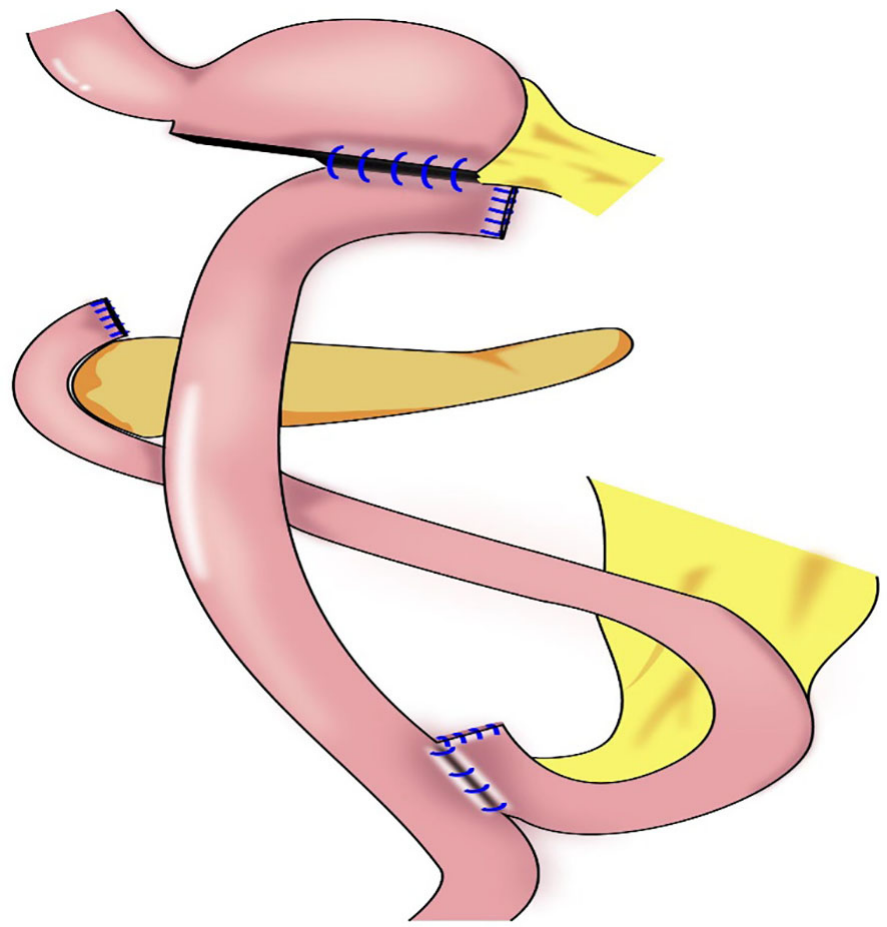
Subtotal gastrectomy: Very small remnant stomach after transection, Roux-en-Y reconstruction procedure were performed.

In 2011, Jiang et al. reported the first use of laparoscopy-assisted subtotal gastrectomy (with a minimal remnant stomach) (**Figure 8**) to treat early upper gastric cancer (53). During the procedure, 1–2 short gastric vessels near the cardia and the left inferior phrenic artery are preserved, and the distal stomach is dissociated approximately 2 cm from the tumor (53). In 2014, Toshiyuki et al. analyzed the feasibility of laparoscopy-assisted subtotal gastrectomy and the nutritional status of patients (54). The authors found that the incidence of postoperative anastomosis-related complications of laparoscopy-assisted subtotal gastrectomy was significantly lower than that with laparoscopy-assisted total gastrectomy, and that weight gain 12 months postoperatively was significantly higher, there were none of recurrence in distant organs, remnant stomach, or lymph nodes with mean follow-up of 27.9 months (54). Souya et al. found that the serum protein concentration and the anti-esophageal reflux effect after subtotal gastrectomy were better compared with those after proximal gastrectomy, and that the hemoglobin concentration was better with subtotal gastrectomy than that with total gastrectomy (55). Itaru et al. reported that body weight and hemoglobin concentrations decreased slightly after laparoscopic subtotal gastrectomy for early upper gastric cancer compared with those after laparoscopic distal gastrectomy, and no difference in total protein and albumin concentrations was noted between the two groups (56). Hao et al. found that patients undergoing laparoscopic-assisted tailored subtotal gastrectomy for advanced gastric cancer in the middle third of the stomach had significantly lower postoperative complication rates (4.2%) compared with patients who underwent laparoscopic assisted total gastrectomy (17.8%). Furthermore, albumin, prealbumin, total protein, and hemoglobin concentrations, and red blood cell counts in the laparoscopic-assisted tailored subtotal gastrectomy group were significantly higher than the related values in the laparoscopic total gastrectomy group, The 3-year overall survival rates in the laparoscopic-assisted tailored subtotal gastrectomy and laparoscopic assisted total gastrectomy groups were 85.6% and 67.4%, respectively (P<0.05) (57). Jin and Liu et al. subsequently designed laparoscopic tailored subtotal gastrectomy (LTSG) to treat advanced middle gastric cancer. On the basis of the premise of guaranteeing tumor safety, tailored resection was performed according to the tumor site to retain as much stomach volume as possible. The main operation points with LTSG are to reserve 1–2 short gastric vessels without No. 2 lymph node dissection, and to ensure upper, lower, and lateral margins of > 3 cm. If the above requirements cannot be met, total gastrectomy should be performed. The study showed that the LTSG group had fewer postoperative complications, better nutritional status, no increased recurrence rate, and a long-term survival benefit compared with total gastrectomy, possibly achieved by improving nutritional status and, thereby, prolonging the patients’ overall survival (57, 58). Itaru et al. analyzed the efficacy of laparoscopic subtotal gastrectomy and laparoscopic distal gastrectomy (56). After 3 years of follow-up, the authors found that body weight and hemoglobin concentration in the laparoscopic subtotal gastrectomy group were lower than the values in the laparoscopic distal gastrectomy group; however, no difference in total protein and albumin concentrations was noted between the two groups (56).

The indications for laparoscopic subtotal gastrectomy comprise a tumor in the upper stomach or invading the upper stomach, preoperative stage cT1N, tumor located < 5 cm from the gastroesophageal junction or < 3 cm from the cut end of the remnant stomach, and negative incision margin (55). At least 1–2 short gastric vessels and posterior gastric vessels from the cardia should be preserved during subtotal gastrectomy. Blood flow to the remnant stomach is mainly supplied by the left inferior phrenic artery and 1–2 short gastric and posterior gastric vessels (54, 57). Subtotal gastrectomy performed after complete dissection of the No. 1 and No. 2 lymph nodes may result in a lack of blood supply to the remnant stomach, worsened motility disorders in the remnant stomach, and poor anastomosis healing.

### 3.5 Cardia-preserving radical gastrectomy

**Figure 9:**
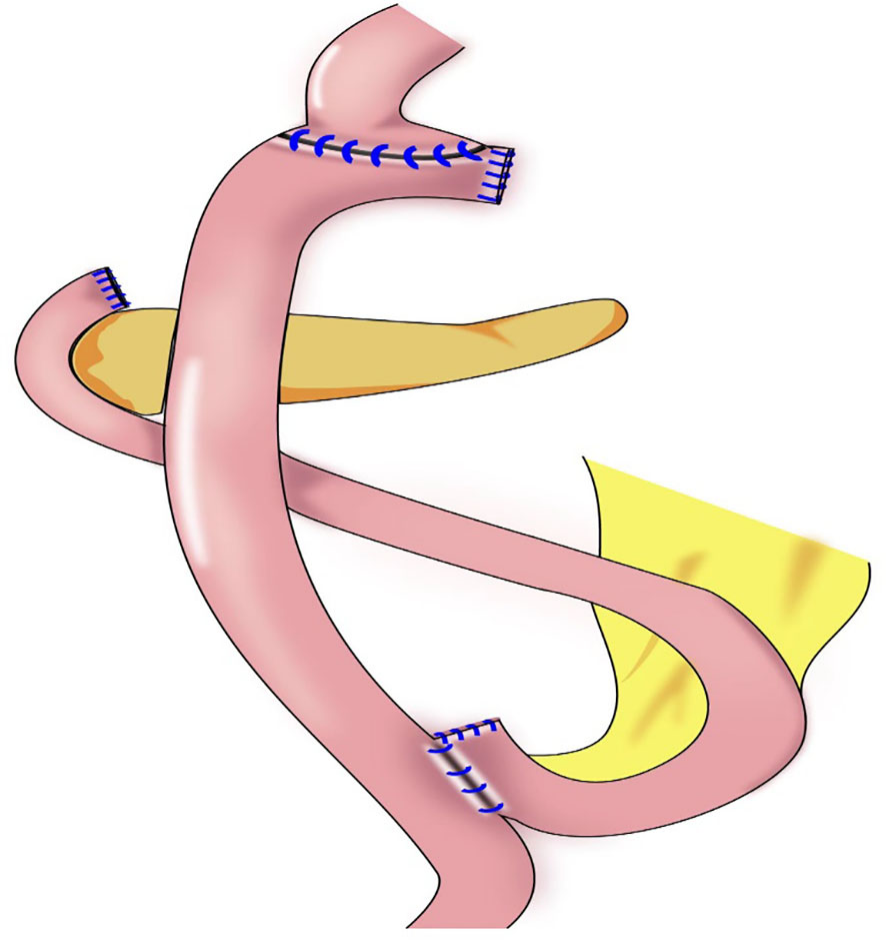
Cardia-preserving radical gastrectomy: The remnant stomach is dissociated 2–3 cm from the cardia using a linear cutting stapler, Roux-en-Y reconstruction procedure were performed.

Our team began to perform cardia-preserving radical gastrectomy (**Figure 9**) in November 2020. No.s 1, 2, 3, 4, 5, 6, 7, 8, and 9 lymph nodes are dissected by laparoscopy; the No. 11 lymph node is dissected along the splenic artery; and one short gastric artery is reserved during dissection of the No. 10 lymph node to complete D2 lymph node dissection. After lymph node dissection is complete, 2–3 cm of the lower esophagus is dissociated, and the remnant stomach is dissociated 2–3 cm from the cardia using a linear cutting stapler. The specimen is extracted through a small incision approximately 4 cm from the umbilicus to determine sufficient incision margins and to confirm negative margins by frozen section. The gastric stump and jejunum are sutured manually and anastomosed (Roux-en-Y). To date, cardia-preserving radical gastrectomy has been performed successfully in 10 cases, without conversion to laparotomy and without severe surgery-related complications, such as postoperative bleeding, anastomotic fistula, or anastomotic stenosis. The proximal and distal margins of the resected specimens were negative in all 10 cases. The patients were followed-up for 2–15 months, with no deaths or tumor recurrence and metastasis during the follow-up period. There were also no postoperative reflux symptoms. Subtotal gastrectomy performed after complete dissection of the No. 1 and No. 2 lymph nodes may result in a lack of blood supply to the remnant stomach, which leads to further motility disorders in the remnant stomach. During cardia-preserving radical gastrectomy, approximately 2–3 cm of the gastric wall away from the dentate line is preserved, with little residual gastric tissue; therefore, the blood supply is relatively better. Complete cardia-preserving radical gastrectomy can reduce the incidence of reflux esophagitis, but its clinical efficacy requires further verification in prospective and controlled clinical studies (59).

The indications for cardia-preserving radical gastrectomy comprise (1) Siewert Type III(2-5cm below the dentate line) for early gastric cancer with the upper edge of the lesion is more than 4 cm from the cardia. (2) Advanced middle gastric cancer, the incision margin is more than 4 cm from the tumor, and the incision margin is at least 2.0-3.0cm below the cardia to ensure the anastomosis distance. (3) Rapid pathological examination should be performed to confirm that the surgical margins was negative.

## 4 Summary and prospects

The gastric cardia has an anti-reflux function, the loss of which significantly increases the incidence of reflux esophagitis and reduces patients’ quality of life. Currently, although various anti-reflux reconstruction methods after proximal gastrectomy reduce the incidence of reflux esophagitis to a certain extent, the incidence is still quite high. The reconstruction process is complicated and postoperative complications increase correspondingly. There are many anti-reflux gastrectomy procedures, but the indications for each procedure are limited.

Cardia-preserving radical gastrectomy has the advantage of more thorough lymph node dissection and wider indications than those for subtotal gastrectomy. However, the clinical efficacy of cardia-preserving radical gastrectomy requires verification in prospective and controlled clinical trials. Cardia-preserving radical gastrectomy is a promising approach as one of the more reasonable anti-reflux surgeries.

## Author contributions

The review was designed and revised by Y-YW, LL, and Y-PM. LL, YR, Z-HL, and Q-TJ conducted the literature collection. X-FC draw the figure and Z-HL, Y-YW, and LL wrote the manuscript. All authors contributed to the article and approved the submitted version.
